# Hypoalbuminemia in hemodialyzed end stage renal disease patients: risk factors and relationships - a 2 year single center study

**DOI:** 10.1186/1471-2369-14-242

**Published:** 2013-11-01

**Authors:** Nagaraja Rao Sridhar, Sowmya Josyula

**Affiliations:** 1 Department of Internal Medicine (Nephrology), Buffalo General Hospital, University at Buffalo, and Buffalo Medical Group, 85 High Street, Buffalo, New York, USA; 2 Research Associate, Kaleida Health (Buffalo General Hospital) 2009. Visiting Observer, Internal Medicine 2008-9, 411, 5th Floor, Kancharla Towers, Golconda X Roads, 500020, Hyderabad, India

## Abstract

**Background:**

Malnutrition affects 1/3 of hemodialysis patients and associates with a higher risk of morbidity and mortality. Serum albumin is a marker of nutrition and inflammation, and predicts mortality, particularly when <3.8 g/dL. This study was performed to identify risk factors for hypoalbuminemia (<3.8 g/dL) and the particular temporal relationship and strength of association between protein intake (nPCR) and serum albumin when confounding variables are taken into account.

**Methods:**

Demographic, clinical, and dialysis-related data of 57 ESRD patients on hemodialysis over 24 months in 6 temporal segments were analyzed with serum albumin as a continuous, and categorical outcome (with 3.8 g/dl as cut-off) variable , against 13 potential independent variables [4 demographic factors, 3 nutrition-related, and 5 morbidity-related parameters, and % urea reduction ratio (URR)]. The temporal relationships between albumin and nPCR were analyzed for the concurrent & 3 subsequent months in each temporal segment.

**Results:**

The impact of nPCR on serum albumin (p < 0.05) was significant but with no discernible temporal relationship. Advancing age, longer vintage, female gender, diabetes mellitus, nPCR, serum phosphate and ferritin had significant correlation with albumin <3.8 g/dl (p < 0.05). Serum phosphate levels correlated positively, and fever, bacteremia, hospital stay and weight loss negatively, with mean serum albumin but did not negate the effect of nPCR. Regression analysis showed that *mean* albumin associated with nPCR, fever, hospital stay, bacteremia, dialysis vintage, age, sex, and diabetes mellitus; and that an albumin level of <3.8 g/dl associated with age, female sex, diabetes, lower nPCR, and higher ferritin.

**Conclusion:**

Suggested target albumin levels were not met in elderly, female, and diabetic patients. The association of nPCR with albumin was not nullified by confounding demographic or morbidity-related factors. nPCR had no demonstrable temporal relationship with albumin.

## Background

370,274 U.S residents received maintenance hemodialysis in 2009 [1]. The expected life span is 20 – 25 years shorter for dialyzed patients than for age, sex, and race – matched U.S. controls over 45 years of age [2]. Protein-energy malnutrition is a major risk factor for mortality and inflammation; the presence of co-morbid conditions like cardiovascular disease increases this risk further [3-7].

Serum albumin is a marker of nutrition and inflammation and predicts mortality [8-10]. Studies have suggested that a serum albumin level of less than 3.8 g/dL (and/or a reduction in serum albumin levels) confers a greater mortality risk in patients with end-stage renal disease (ESRD) [11,12] and in various other disease states [13]. Guidelines proposed by the Centers for Medicare and Medicaid Services (CMS), Dialysis Outcomes Quality Initiative (DOQI) and the International Society of Nutrition Managers to maintain target serum albumin levels in ESRD are >/= 3.5, 4.0, and 3.8 g/dl respectively [10,14,15]. Serum albumin levels are considered indicators of quality of care at some dialysis facilities and may be included as a parameter of quality by the Centers for Medicare and Medicaid Services (CMS) in the future. A large study of intra-dialytic parenteral nutrition (IDPN) did not show a mortality benefit vis-à-vis oral supplements [16]. In the present retrospective study, we tried to identify factors associated with serum albumin and identify risk factors for hypoalbuminemia (< 3.8 g/dL) using only demographic and those biochemical measurements that are routinely available in the United States; we were particularly interested in the temporal association between nPCR (normalized protein catabolic rate) as an indicator of protein intake and serum albumin over an extended period of 24 consecutive months. Further, we tried to understand whether this relationship between nPCR and albumin was robust when the confounding effects of other factors that appeared to correlate with albumin levels in univariate analyses were taken into account.

## Methods

The current study was undertaken at a single tertiary-care hospital-based outpatient hemodialysis center with about 100 patients during the study period of which regular, uninterrupted, serum albumin levels and 10 other numerical variables were available for 57 patients for 24 consecutive months (the remaining patients were transferred to free-standing centers within a few months, died, or received transplants) who gave signed informed consent to have their data included in the study which was approved by the University at Buffalo’s Institutional Review Board. The inclusion criteria included patients who were on maintenance hemodialysis from February 2008 to February 2010 and their ability to sign an informed consent. Hemodialysis was performed three times weekly in 55 patients and twice weekly in 2 patients. Low flux dialyzers using modified cellulosic membranes were used in all the patients. There were no major changes made in the dialysis prescription during the two year period except those pertaining to target weight, heparin, and dialysate potassium concentration.

All patients had been advised to restrict total dietary phosphate to between 1000 and 1500 mg per day. Food diaries were not available for review.

A total of 10 patients had been dialyzed at this tertiary hospital-based outpatient dialysis facility for longer than 4 months but less than 24 months and were not included.

The outcome variable, serum albumin, was measured by the bromocresol purple method which gives slightly lower values than the bromocresol green method. Therefore a value of 3.8 g/dl which is 0.2 g/dl lower than that suggested by K/DOQI was used for analysis; this is 0.3 g/dl higher than the target suggested by CMS. Age, dialysis vintage in months, nPCR, serum phosphate, % dry weight change, body temperature before, during, and at closure of HD to enumerate the number of febrile episodes if >37 degrees C, the number of episodes of bacteremia, the number of days spent at any hospital when the outpatient status of the patient was suspended, and the % URR were used as continuous independent variables. Patient-gender and diabetes mellitus were used as categorical independent variables. All the laboratory measurements were made by standard assay methods at the clinical laboratories of Kaleida Health. Dialysis vintage was counted as months from the commencement of any form of dialysis. All numerical data were expressed in unaltered form over the 2 years and as cross-sectional means for evaluation of temporal relationships within each 4-month period over the 2 years (58 variables × 57 patients × 24 months).

### Statistical analysis

The analysis was done using PASW/18 (2009 SPSS Inc.) and the variables expressed as means ± SD. p < 0.05 was considered to be significant. The relationship between serum albumin as a continuous (scaled) variable and each of the independent numerical variables was studied using the Pearson Correlation Coefficient; the strength of this association was then tested using multiple regression including other covariates and the attributable impact of each independent variable on mean serum albumin levels was calculated as the coefficient beta (standardized for the varied units used to measure the 11 non-demographic variables) with 95% confidence intervals (CI). A 2 × 2 table was used to study the impact of gender and diabetes mellitus on the number of instances of albumin < 3.8 g/dl by calculating the likelihood ratio using Chi-square tests with continuity correction. Binary Logistic Regression (BLR) was used for multi variable relationships with serum albumin levels of <3.8 g/dl as the categorical outcome variable and the likelihood ratio of each independent variable (exponent B) derived with 95% CI; the significance of the results was calculated with and without bootstrapping of 1000 sample iterations across variables in an effort to make the analysis robust. Temporal relationships between serum albumin and nPCR (as a measure of protein intake) and serum ferritin (which correlates negatively with serum albumin) were studied using Pearson’s correlation with data submitted to analysis as continuous and 4-monthly cross-sectional means respectively; the 24 month period was divided into 6 blocks of 4 months each because serum ferritin was measured only once every 4 months by unit policy necessitated by CMS reimbursement. Erythrocyte sedimentation rates and C-reactive protein levels were not routinely available for the 57 patients over the 24 months studied, again determined by standard reimbursement related policies. The specific impact of a given month’s mean nPCR on the mean serum albumin levels of the concurrent and 3 subsequent months was expressed as the F statistic with 3 degrees of freedom for 4 methods of statistical analysis: Pillai’s trace, Wilk’s lambda, Hoteling’s trace and Roy’s largest root. Finally, multinomial regression was used to study the impact of each factor shown to be significant by Pearson correlation on a range of outcome albumin levels to double check the impact of nPCR, the independent variable of interest, at each cut-off point of albumin from 1.9 through 4.1 g/dl.

## Results

Of the 57 patients 26 were male and 31 were female. The median age was 57 years (range 29 to 85 years) and the mean 58 years. The causes of renal failure included the following: diabetic nephropathy (n = 27), primary hypertensive renal disease (n = 17), chronic glomerulonephritis (n = 9), unknown etiology (n = 7) and obstructive pyelonephritis (n = 1).

The mean values of the clinical and dialysis data, serum biochemistry results, and potential contributors to hypoalbuminemia of 57 patients are given in Table 1.

**Table 1:** Clinical, dialysis and laboratory data in 57 hemodialysis patients and co-morbid features predisposing to hypoalbuminemia

**Parameter**	**Mean ± SD**
Age (yrs)	57.9 ± 14.6
Dialysis vintage (mo)	47.8 ± 38.6
BMI (kg/sq.m)	30.2 ± 10.9
Albumin(g/dl)	3.38 ± 0.40
Ferritin (ng/ml)	623.57 ± 356.04
nPCR	1.01 ± 0.33
Phosphate (mg/dl)	5.69 ± 1.91
URR%	73.96 ± 19.99
WBC (1000/cmm)	6.72 ± 2.32
***Comorbid Features***	*n*
Catheter use (6–24 months)	18
Coronary disease	12
Peripheral vascular disease	08
CHF (systolic +/- diastolic)	10
CHF (diastolic alone)	06
Malignancy	05

### Univariate analysis

**Figure 1:**
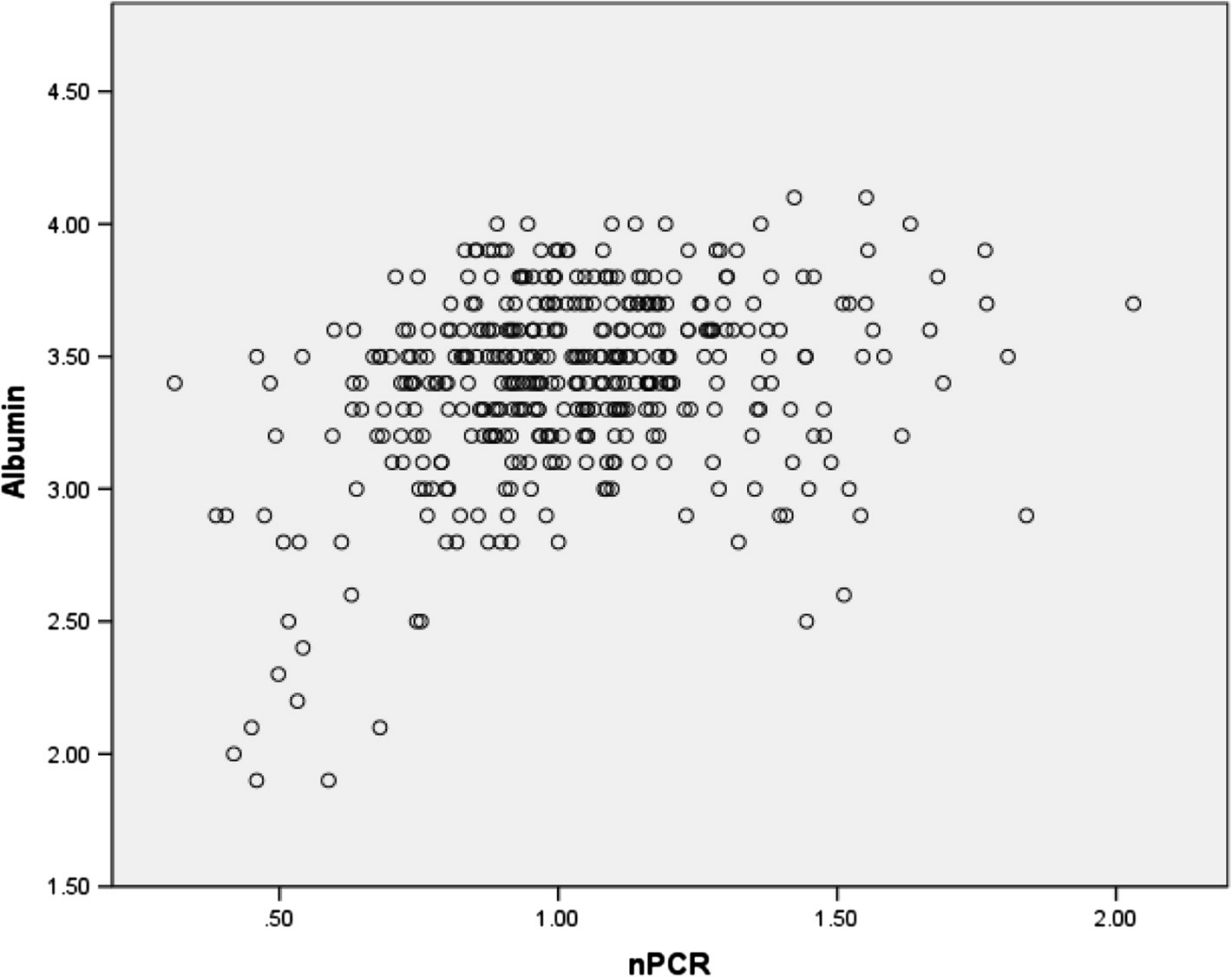
Serum albumin by gender and decile of age.

**Figure 2:**
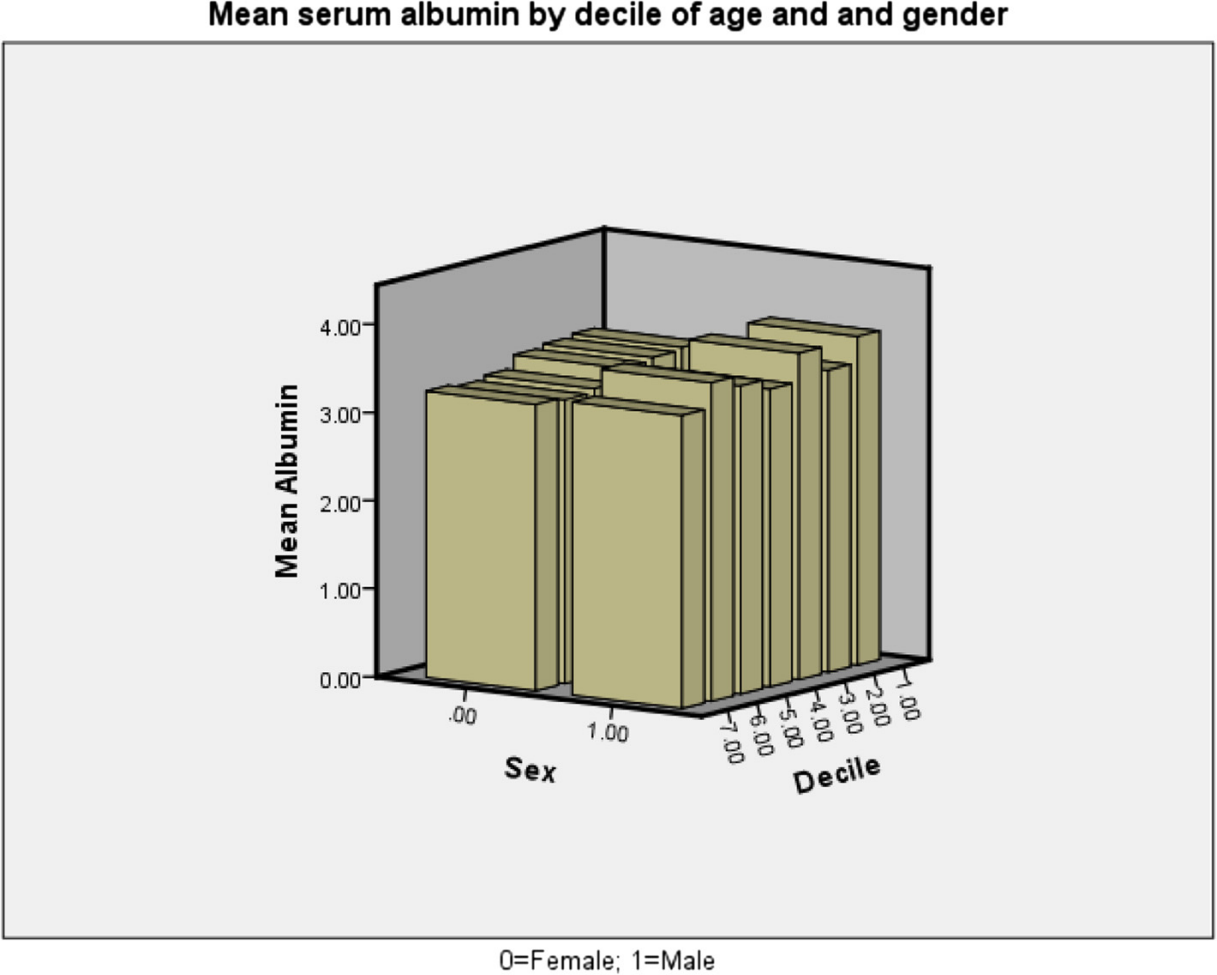
Serum albumin by age decile and diabetic status.

**Figure 3:**
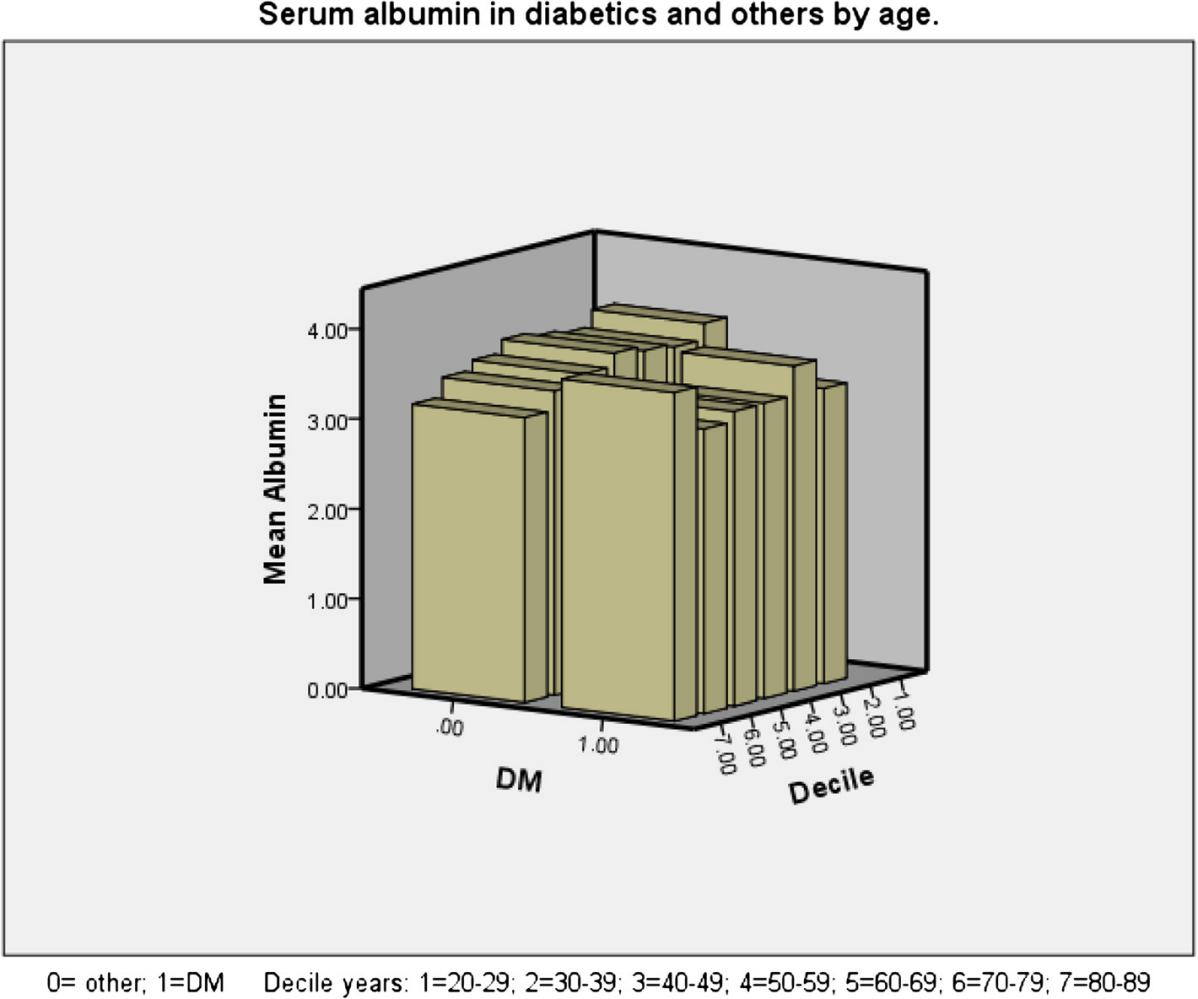
Relationship between nPCR and mean serum albumin.

An inverse correlation was found between age and mean serum albumin (p < 0.001). Advancing age associated with lower albumin (especially in women (Figure 1) and in both diabetics and non-diabetics (Figure 2) except in the 9th decade in which there were only 5 diabetics who had a higher mean albumin level than their 18 non-diabetic counterparts among our patients, mainly due to the values of 2 patients. Of the continuous variables nPCR (Figure 3), % change in post-HD goal (dry) weight, serum phosphate were positively correlated with mean serum albumin levels (p < 0.001 for all) whereas the number of episodes of bacteremia (p < 0.001), febrile episodes, and days spent in hospital (p < 0.05 for both) showed a negative correlation with mean albumin levels. Dialysis vintage, % URR, and ferritin did not show a correlation with mean albumin (Table 2). Female and diabetic patients were more likely to have a serum albumin <3.8 g/dl (Table 3).

**Table 2:** Correlation between mean 4 monthly serum albumin and risk factors

	**Pearson coefficient**	***p***
Age in years	-0.25	<0.001
Vintage in months	0.05	0.35
nPCR	0.31	<0.001
% Goal Weight Change	0.23	<0.001
Serum Phosphate (mg/dl)	0.22	<0.001
%URR	0.03	0.55
Episodes of fever	-0.11	0.02
Leukocytes /cmm	-0.09	0.07
Ferritin (ng/ml)	0.02	0.64
Bacteremia (episodes)	-0.27	<0.001
Days in hospital	-0.13	0.01

P < 0.05 = significant.

**Table 3:** 2 x 2 Table for risk of hypoalbuminemia by gender and diabetes

		**Alb >3.8 g/dl Instances (%)**	**Alb <3.8 g/dl Instances (%)**	**Total (%)**
Gender (*)	Female	13 (7)	173 (93)	186 (100)
	Male	35 (22.4)	121 (77.6)	156 (100)
Diabetes(#)	Present	6 (3.6)	161 (96.4)	167 (100)
	Absent	42 (24)	133 (76)	175 (100)

* Likelihood ratio (Chi-square with continuity correction) of instances of albumin <3.8 in female patients = 17.08 (asymptotic *p* < 0.001).

# Likelihood ratio (Chi-square with continuity correction) of instances of albumin <3.8 g/dl in diabetics = 27.8 (asymptotic *p* < 0.001).

### Multivariate analysis

Multiple linear regression (Table 4) showed that mean serum albumin as the outcome variable was most significantly impacted by age, diabetes mellitus, hospital stay, and bacteremia (p</=0.005); and to a smaller degree of significance by gender , vintage , and nPCR (p < 0.05 for each). Binary logistic regression (BLR) showed that advanced age, female sex, and diabetes mellitus conferred the greatest hazard of a serum albumin <3.8 g/dl (p</=0.008) followed by nPCR, dialysis vintage, and ferritin (p < 0.05). Serum phosphate and %URR did not appear to impact either the mean or the target albumin levels by BLR (Table 5).

**Table 4:** Results of multiple linear regression: effect of risk factors on mean serum albumin

**Model**	**Standardized Coefficient**	**95% CI**	**95% CI**	**2-tailed *p***
	(Beta)	Lower	Upper	
(Constant)	3.277	2.713	3.728	0.001 *
nPCR	0.180	0.063	0.415	0.010 *
URR% (Mean)	0.048	-0.003	0.010	0.413
Ferritin (ng/ml)	-0.038	-5.197	0.000	0.395
Phosphate (mg/dl)	0.043	-0.011	0.033	0.376
WBC x 1000/cmm	-0.044	-0.024	0.009	0.372
Weight change%	0.043	-0.006	0.012	0.458
Fever	0.097	-0.013	0.138	0.036 *
Days in Hospital	-0.301	-0.022	-0.007	0.001 *
Bacteremia Episodes	-0.179	-0.286	-0.035	0.005 *
Vintage (months)	-0.100	-0.002	-5.187	0.035 *
Male Gender	0.105	0.005	0.146	0.044 *
Diabetes mellitus	-0.163	-0.185	-0.046	0.004 *
Age in years	-0.175	-0.006	-0.002	0.001 *

* = Significant.

**Table 5:** Results of binary logistic regression: serum albumin < 3.8 vs. risk factors

		**95%**	**CI**		
Factor	LR (exp B)	Lower	Upper	*p*	*p* (with bootstrapping)
Age in years	1.029	1.000	1.059	0.051 *	0.008 *
Female Sex	3.684	1.566	8.667	0.003 *	0.001 *
Diabetes mellitus	0.137	0.049	0.381	<0.001 *	0.001 *
Vintage (months)	0.992	0.982	1.002	0.103	0.057
%URR	1.006	0.927	1.092	0.878	0.870
nPCR	0.232	0.051	1.045	0.057	0.043 *
Serum Phosphate	0.912	0.689	1.208	0.521	0.447
%Weight Loss	1.082	0.980	1.194	0.119	0.128
Days in Hospital	1.042	0.926	1.174	0.492	0.470
Febrile Episodes	1.502	0.413	5.456	0.537	0.501
Bacteremia	0.982	0.248	3.891	0.979	0.962
Leukocytosis	0.955	0.766	1.190	0.682	0.696
Serum Ferritin	0.999	0.997	1.000	0.064	0.047 *

* = significant. LR = Likelihood Ratio. Bootstrapping performed with 1000 iterations.

The albumin levels of any 4 consecutive months did not significantly differ from each other or the index month by ANOVA. The effect of nPCR of the index month on mean albumin in any of the four subsequent months was not significantly different than on that of the index month: the exact statistic (F) for albumin being 0.572 for each of the 4 months in any temporal segment (p = 0.63) and the within-subjects-design (intercept) against nPCR of month 0 (index month) for each segment was 0.641 (p = 0.58). Thus we were not able to demonstrate any longitudinal pattern of variation in the albumin levels relative to nPCR of any given month.

Multinomial regression demonstrated the impact of nPCR at each level of increment of albumin of 0.1 g/dl from 1.9 to 4.1 g/dl when fever, days in hospital, bacteremia, and dry weight change, age (decile), sex, phosphorus, and WBC counts, the factors shown by Pearson correlation to be significant or nearly significant, were taken into account (p < 0.001).

## Discussion

This retrospective analysis suggests that the suggested serum albumin targets were not met in the elderly and diabetic patients, particularly women and that there was a significant association between mean albumin and nPCR (protein intake) which was not nullified by confounding non-nutritional factors. Such hypoalbuminemia is known to unfavorably alter prognosis [17-20].

Recent publications have confirmed an association of serum albumin and mortality though some have questioned its value as a nutritional marker [11,12,15,21] because acute illnesses, especially inflammation, appear to be associated with a reduction in its levels, most likely due to catabolism [22]. Hypoalbuminemia is associated with hypotension [23], progressive left ventricular dysfunction [24] and reduced intra-dialytic skin blood flow [25] and is due to decreased synthesis; attempts to correct hypoalbuminemia have not resulted in improved mortality so far [26-37].

Age, sex, catheter use, nPCR, creatinine, dialysis adequacy, WBC number, neutrophil:lymphocyte ratio, C-reactive protein, hemoglobin and aspartate aminotransferase(AST) level are associated with albumin levels in the serum [27-29]. Advanced age (>65 years) is associated with lower serum albumin in large cross-sectional studies [38,39]. We used age as a continuous and ordinal variable and found a correlation with hypoalbuminemia in both men and women (Tables 2). Reductions in dietary protein and energy intake appear to be the likely reasons for hypoalbuminemia in the elderly [40,41]. Our results confirm a direct, and somewhat robust association between protein intake (nPCR) and serum albumin similar to the results of others [42-45] . Investigators studying a small series of patients found a catabolic state when the dietary protein intake was 42.5 g/d (nPCR, 0.78 g/kg/d) and an anabolic state when the dietary protein intake was 73.5 g/d (nPCR, 0.88 g/Kg/d) in a prospective cross-over trial confirming an association between dietary protein intake and nPCR in hemodialyzed patients [46]; the MDRD study in pre-ESRD patients showed similar results [47]. As described in the literature [48], advancing age, female sex, and the presence of diabetes were associated with a greater hazard of hypoalbuminemia in our patients. Unlike some investigators [49] we did not find a stronger relationship between albumin and indicators of inflammation (ferritin, WBC, fever) than between albumin and protein intake (nPCR) and serum phosphate. The relationship of albumin and nPCR remained highly significant after discounting the effect of phosphate levels in our group of patients using partial correlation (p < 0.001) and multi-variable methods (p = 0.01) consistent with the experience of other investigators [50]. Mortality is associated with lower serum albumin levels and nPCR in well dialyzed patients [51,52] and dietary phosphate intake and dietary phosphate to protein ratio have been shown to have an adverse effect on mortality in a recent study [53]. Zitt et al. in a prospective cohort study (INVOR) found a higher risk of mortality when the albumin and phosphate were both low or high than when the phosphate levels were low and those of albumin high [54]. However, prescribed dietary phosphate restriction was associated with higher mortality when time-dependent confounders were included in a post hoc analysis of the Hemodialysis Study whose authors postulate that phosphate-rich foods tend to be nutrient dense and presumably protein-rich [55]. Serum phosphate levels may reflect this greater nutrient intake although adherence to phosphate binders and the degree of hyperparathyroidism would also impact them. A surprising lack of association was noticeable between serum albumin and small solute clearance as indicated by % URR in our patients. We attribute this finding to our unit policy that dictated the use of dialyzers with higher urea clearance, increasing dialysis time and blood flow rates when KT/V was <1.4 which resulted in few instances of under-dialysis. We used URR % (which is mandated by CMS) because many dialysis facilities do not routinely calculate KT/V and the latter is intrinsically related to nPCR. A lack of association of weight loss (as indicated by changes in the post dialysis goal weight) with serum albumin levels <3.8 g/dl was seen due to an insufficient number of instances of weight loss during the study period; however, there was a correlation between such goal weight changes and *mean* serum albumin (Tables 2 &4). Dry weight is determined by nephrologists by clinical trial and error with the attendant pitfalls when used for analysis. We did not find an association between baseline dry weight and albumin or inflammatory markers [56,57]. We did not analyze inter-dialytic weight gain vs. albumin change in our study; others have observed a negative effect of lower interdialytic weight gain on nPCR, prealbumin, and dry weight in longitudinal analyses [57]. Dialysis vintage is associated with a higher relative risk of death in case-mix adjusted analyses independent of nutritional and other variables in larger cohorts [58]. Vintage appeared to be associated with albumin < 3.8 g/dl in our patients only by univariate methods, and was of borderline significance in multivariate analyses, but did not correlate with mean serum albumin. Inflammation and dietary protein intake are known to impact serum albumin in opposing ways [59]. One effect of the chronic inflammatory state in dialysis patients is sustained hypoalbuminemia according to the HEMO Study [60]. Serum ferritin is measured routinely in most U.S dialysis units to guide intravenous iron therapy, and is a surrogate for inflammation that associates with short term mortality and hypoalbuminemia [61]; it appeared to correlate with albumin <3.8 g/dl in our study. Hospitalization is associated with malnutrition in general, and protein malnutrition in particular, even in well dialyzed patients [53,62] and correlates with weight loss, reduction in albumin, and inflammation, particularly when the stay is prolonged and caused by inflammatory conditions [63]; we did not find a correlation of hospital stay with albumin <3.8 g/dl but it was negatively associated with mean albumin levels. The bromocresol purple method of albumin measurement which gives slightly lower levels is closer to the gold standard of immunonephelometry and may provide value to our data [64,65].

### Limitations of the study

This was a retrospective analysis of patient data with the attendant pitfalls. Residual renal function (which can lower nPCR) was not studied because there were only a few patients who were not oliguric and 24 hour urine determinations were not available. Such residual function would likely inflate, not diminish, the impact of nPCR on albumin. We were not able to demonstrate any longitudinal pattern of variation in the albumin levels relative to nPCR of any given month. Also, observation of the data suggested a relatively small number of instances of hospital stay and positive blood culture; this could have diminished the demonstrated impact of inflammation. C-reactive protein and pre-albumin levels would have provided valuable information and were not available and are not generally measured in U.S dialysis units routinely.

## Conclusions

Protein intake as measured by nPCR correlates with serum albumin even when confounding factors are taken into account. Prospective studies are required to confirm whether albumin targets would be difficult to achieve in elderly female diabetics and whether protein supplementation would alter albumin levels in such vulnerable subgroups.

## Competing interests

The authors declare that they have no competing interests.

## Authors’ contributions

NS conceived of and planned the study, performed statistical analyses, prepared tables and graphs, wrote the abstract and manuscript, mentored SJ in collation of data, and corresponded with the publisher. SJ collected and collated the data, obtained informed consent from patients for inclusion of their data, obtained Institutional Review Board approval, compiled and assigned references quoted in the paper. Both authors read and approved the final manuscript.

## Pre-publication history

The pre-publication history for this paper can be accessed here:

http://www.biomedcentral.com/1471-2369/14/242/prepub
